# Women’s Perceptions and Experiences of Breastfeeding: a scoping review of the literature

**DOI:** 10.1186/s12889-021-12216-3

**Published:** 2021-11-26

**Authors:** Bridget Beggs, Liza Koshy, Elena Neiterman

**Affiliations:** School of Public Health Sciences, University of Waterloo, 200 University Ave West, Waterloo, ON N2L 3G1 Canada

**Keywords:** Breastfeeding, Experiences, Public health, Review, Women

## Abstract

**Background:**

Despite public health efforts to promote breastfeeding, global rates of breastfeeding continue to trail behind the goals identified by the World Health Organization. While the literature exploring breastfeeding beliefs and practices is growing, it offers various and sometimes conflicting explanations regarding women’s attitudes towards and experiences of breastfeeding. This research explores existing empirical literature regarding women’s perceptions about and experiences with breastfeeding. The overall goal of this research is to identify what barriers mothers face when attempting to breastfeed and what supports they need to guide their breastfeeding choices.

**Methods:**

This paper uses a scoping review methodology developed by Arksey and O’Malley. PubMed, CINAHL, Sociological Abstracts, and PsychInfo databases were searched utilizing a predetermined string of keywords. After removing duplicates, papers published in 2010–2020 in English were screened for eligibility. A literature extraction tool and thematic analysis were used to code and analyze the data.

**Results:**

In total, 59 papers were included in the review. Thematic analysis showed that mothers tend to assume that breastfeeding will be easy and find it difficult to cope with breastfeeding challenges. A lack of partner support and social networks, as well as advice from health care professionals, play critical roles in women’s decision to breastfeed.

**Conclusion:**

While breastfeeding mothers are generally aware of the benefits of breastfeeding, they experience barriers at individual, interpersonal, and organizational levels. It is important to acknowledge that breastfeeding is associated with challenges and provide adequate supports for mothers so that their experiences can be improved, and breastfeeding rates can reach those identified by the World Health Organization.

## Background

Public health efforts to educate parents about the importance of breastfeeding can be dated back to the early twentieth century [1]. The World Health Organization is aiming to have at least half of all the mothers worldwide exclusively breastfeeding their infants in the first 6 months of life by the year 2025 [2], but it is unlikely that this goal will be achieved. Only 38% of the global infant population is exclusively breastfed between 0 and 6 months of life [2], even though breastfeeding initiation rates have shown steady growth globally [3]. The literature suggests that while many mothers intend to breastfeed and even make an attempt at initiation, they do not always maintain exclusive breastfeeding for the first 6 months of life [4, 5]. The literature identifies various barriers, including return to paid employment [6, 7], lack of support from health care providers and significant others [8, 9], and physical challenges [9] as potential factors that can explain premature cessation of breastfeeding.

From a public health perspective, the health benefits of breastfeeding are paramount for both mother and infant [10, 11]. Globally, new mothers following breastfeeding recommendations could prevent 974,956 cases of childhood obesity, 27,069 cases of mortality from breast cancer, and 13,644 deaths from ovarian cancer per year [11]. Global economic loss due to cognitive deficiencies resulting from cessation of breastfeeding has been calculated to be approximately USD $285.39 billion dollars annually [11]. Evidently, increasing exclusive breastfeeding rates is an important task for improving population health outcomes. While public health campaigns targeting pregnant women and new mothers have been successful in promoting breastfeeding, they also have been perceived as too aggressive [12] and failing to consider various structural and personal barriers that may impact women’s ability to breastfeed [1]. In some cases, public health messaging itself has been identified as a barrier due to its rigid nature and its lack of flexibility in guidelines [13]. Hence, while the literature on women’s perceptions regarding breastfeeding and their experiences with breastfeeding has been growing [14–16], it offers various, and sometimes contradictory, explanations on how and why women initiate and maintain breastfeeding and what role public health messaging plays in women’s decision to breastfeed.

The complex array of the barriers shaping women’s experiences of breastfeeding can be broadly categorized utilizing the socioecological model, which suggests that individuals’ health is a result of the interplay between micro (individual), meso (institutional), and macro (social) factors [17]. Although previous studies have explored barriers and supports to breastfeeding, the majority of articles focus on specific geographic areas (e.g. United States or United Kingdom), workplaces, or communities. In addition, very few articles focus on the analysis of the interplay between various micro, meso, and macro-level factors in shaping women’s experiences of breastfeeding. Synthesizing the growing literature on the experiences of breastfeeding and the factors shaping these experiences, offers researchers and public health professionals an opportunity to examine how various personal and institutional factors shape mothers’ breastfeeding decision-making. This knowledge is needed to identify what can be done to improve breastfeeding rates and make breastfeeding a more positive and meaningful experience for new mothers.

## The review

### Aim

The aim of this scoping review is to synthesize evidence gathered from empirical literature on women’s perceptions about and experiences of breastfeeding. Specifically, the following questions are examined:

1. What does empirical literature report on women’s perceptions on breastfeeding?
2. What barriers do women face when they attempt to initiate or maintain breastfeeding?
3. What supports do women need in order to initiate and/or maintain breastfeeding?

Focusing on women’s experiences, this paper aims to contribute to our understanding of women’s decision-making and behaviours pertaining to breastfeeding. The overarching aim of this review is to translate these findings into actionable strategies that can streamline public health messaging and improve breastfeeding education and supports offered by health care providers working with new mothers.

### Design

This research utilized Arksey & O’Malley’s [18] framework to guide the scoping review process. The scoping review methodology was chosen to explore a breadth of literature on women’s perceptions about and experiences of breastfeeding. A broad research question, “What does empirical literature tell us about women’s experiences of breastfeeding?” was set to guide the literature search process.

### Search methods

The review was undertaken in five steps: (1) identifying the research question, (2) identifying relevant literature, (3) iterative selection of data, (4) charting data, and (5) collating, summarizing, and reporting results. The inclusion criteria were set to empirical articles published between 2010 and 2020 in peer-reviewed journals with a specific focus on women’s self-reported experiences of breastfeeding, as well as how others see women’s experiences of breastfeeding. The focus on women’s perceptions of breastfeeding was used to capture the papers that specifically addressed their experiences and the barriers that they may encounter while breastfeeding. Only articles written in English were included in the review. The keywords utilized in the search strategy were developed in collaboration with a librarian (Table 1). PubMed, CINAHL, Sociological Abstracts, and PsychInfo databases were searched for the empirical literature, yielding a total of 2885 results.

**Table 1:** Search Phrase Utilized For Literature Search

(women AND experiences OR experience OR women’s experiences)	
(breastfeeding OR breast feeding OR lactation OR breast milk)	

### Search outcome

**Fig. 1:**
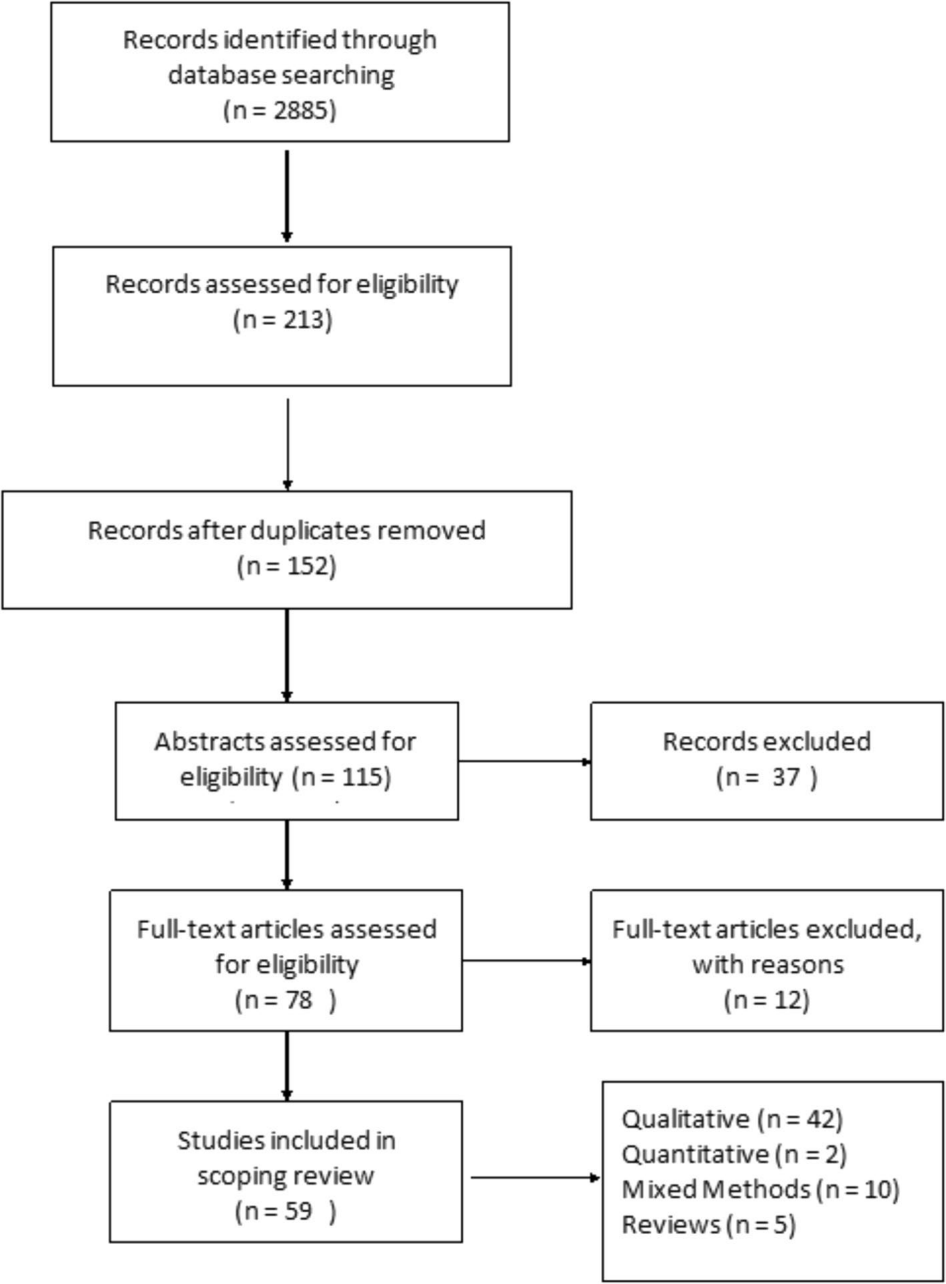
Prisma Flow Diagram

The articles deemed to fit the inclusion criteria (*n* = 213) were imported into RefWorks, an online reference manager tool and further screened for eligibility (Fig. 1). After the removal of 61 duplicates and title/abstract screening, 152 articles were kept for full-text review. Two independent reviewers assessed the papers to evaluate if they met the inclusion criteria of having an explicit analytic focus on women’s experiences of breastfeeding.

### Quality appraisal

Consistent with scoping review methodology [18], the quality of the papers included in the review was not assessed.

### Data abstraction

A literature extraction tool was created in MS Excel 2016. The data extracted from each paper included: (a) authors names, (b) title of the paper, (c) year of publication, (d) study objectives, (e) method used, (f) participant demographics, (g) country where the study was conducted, and (h) key findings from the paper.

### Synthesis

Thematic analysis was utilized to identify key topics covered by the literature. Two reviewers independently read five papers to inductively generate key themes. This process was repeated until the two reviewers reached a consensus on the coding scheme, which was subsequently applied to the remainder of the articles. Key themes were added to the literature extraction tool and each paper was assigned a key theme and sub-themes, if relevant. The themes derived from the analysis were reviewed once again by all three authors when all the papers were coded. In the results section below, the synthesized literature is summarized alongside the key themes identified during the analysis.

## Results

**Fig. 2:**
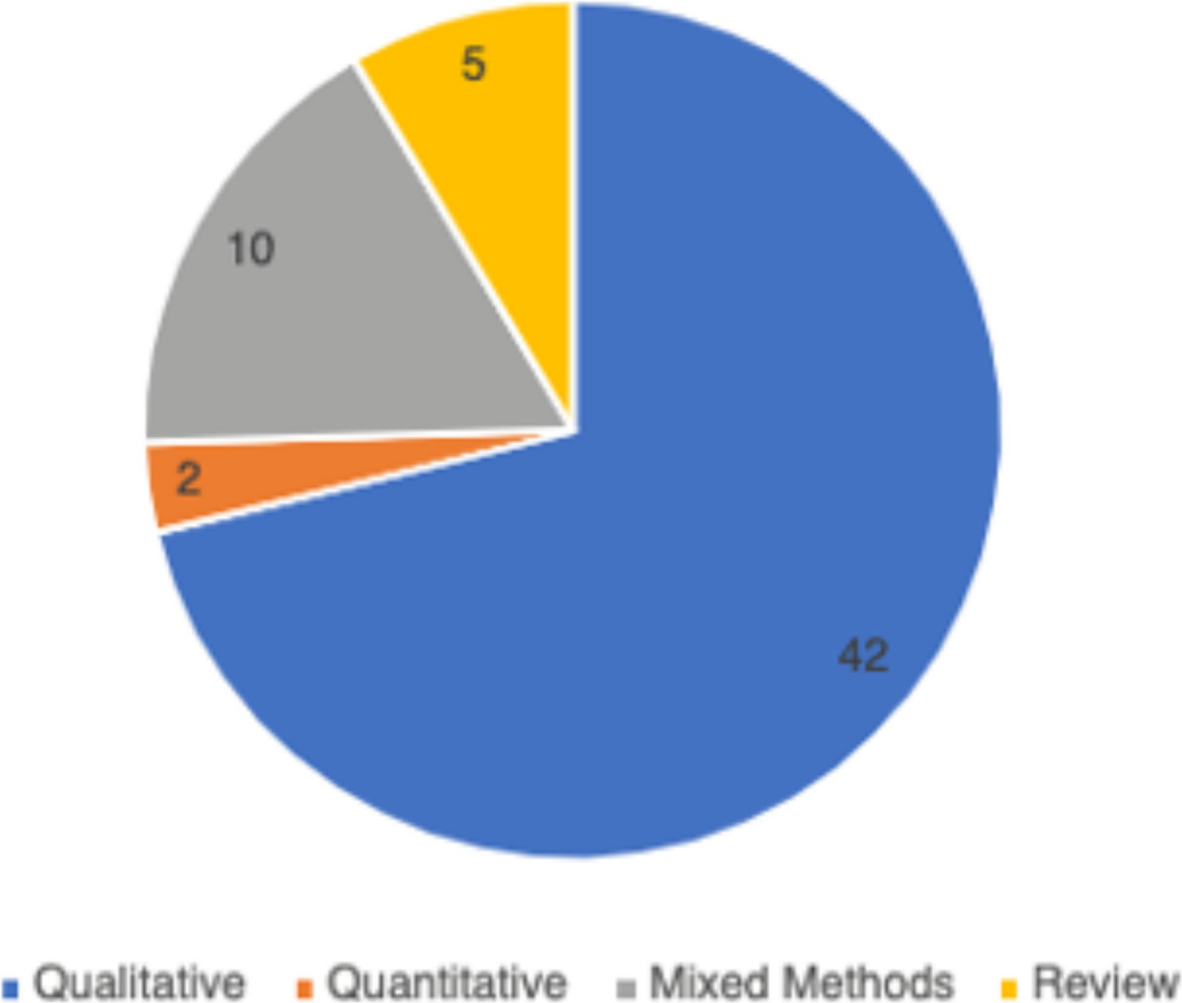
Types of Articles

**Fig. 3:**
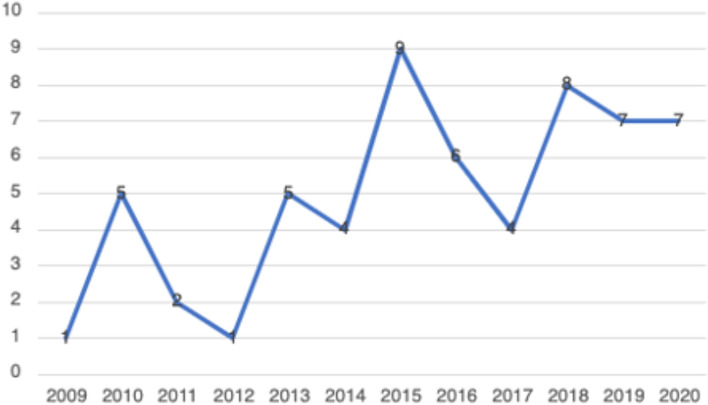
Years of Publication

**Fig. 4:**
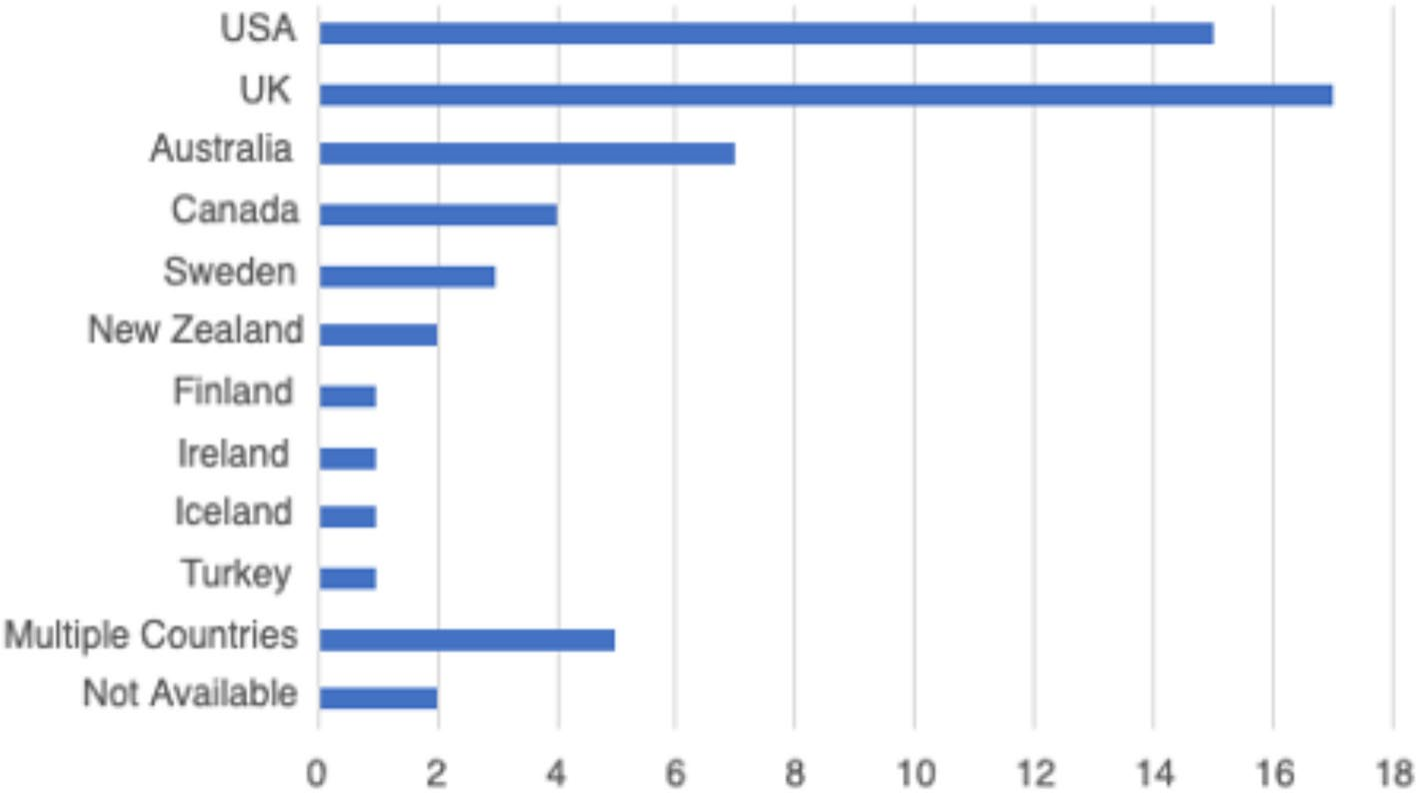
Countries of Focus Examined in Literature Review

In total, 59 peer-reviewed articles were included in the review. Since the review focused on women’s experiences of breastfeeding, as would be expected based on the search criteria, the majority of articles (*n* = 42) included in the sample were qualitative studies, with ten utilizing a mixed method approach (Fig. 2). Figure 3 summarizes the distribution of articles by year of publication and Fig. 4 summarizes the geographic location of the study.

### Perceptions about breastfeeding

Women’s perceptions about breastfeeding were covered in 83% (*n* = 49) of the papers. Most articles (*n* = 31) suggested that women perceived breastfeeding as a positive experience and believed that breastfeeding had many benefits [19, 20]. The phrases “breast is best” and “breastmilk is best” were repeatedly used by the participants of studies included in the reviewed literature [21]. Breastfeeding was seen as improving the emotional bond between the mother and the child [20, 22, 23], strengthening the child’s immune system [24, 25], and providing a booster to the mother’s sense of self [1, 26]. Convenience of breastfeeding (e.g., its availability and low cost) [19, 27] and the role of breastfeeding in weight loss during the postpartum period were mentioned in the literature as other factors that positively shape mothers’ perceptions about breastfeeding [28, 29].

The literature suggested that women’s perceptions of breastfeeding and feeding choices were also shaped by the advice of healthcare providers [30, 31]. Paradoxically, messages about the importance and relative simplicity of breastfeeding may also contribute to misalignment between women’s expectations and the actual experiences of breastfeeding [32]. For instance, studies published in Canada and Sweden reported that women expected breastfeeding to occur “naturally”, to be easy and enjoyable [23]. Consequently, some women felt unprepared for the challenges associated with initiation or maintenance of breastfeeding [31, 33]. The literature pointed out that mothers may feel overwhelmed by the frequency of infant feedings [26] and the amount as well as intensity of physical difficulties associated with breastfeeding initiation [33]. Researchers suggested that since many women see breastfeeding as a sign of being a “good” mother, their inability to breastfeed may trigger feelings of personal failure [22, 34].

Women’s personal experiences with and perceptions about breastfeeding were also influenced by the cultural pressure to breastfeed. Welsh mothers interviewed in the UK, for instance, revealed that they were faced with judgement and disapproval when people around them discovered they opted out of breastfeeding [35]. Women recalled the experiences of being questioned by others, including strangers, when they were bottle feeding their infants [9, 35, 36].

### Barriers to breastfeeding

The vast majority (*n* = 50) of the reviewed literature identified various barriers for successful breastfeeding. A sizeable proportion of literature (41%, *n* = 24) explored women’s experiences with the physical aspects of breastfeeding [23, 33]. In particular, problems with latching and the pain associated with breastfeeding were commonly cited as barriers for women to initiate breastfeeding [23, 28, 37]. Inadequate milk supply, both actual and perceived, was mentioned as another barrier for initiation and maintenance of breastfeeding [33, 37]. Breastfeeding mothers were sometimes unable to determine how much milk their infants consumed (as opposed to seeing how much milk the infant had when bottle feeding), which caused them to feel anxious and uncertain about scheduling infant feedings [28, 37]. Women’s inability to overcome these barriers was linked by some researchers to low self-efficacy among mothers, as well as feeling overwhelmed or suffering from postpartum depression [38, 39].

In addition to personal and physical challenges experienced by mothers who were planning to breastfeed, the literature also highlighted the importance of social environment as a potential barrier to breastfeeding. Mothers’ personal networks were identified as a key factor in shaping their breastfeeding behaviours in 43 (73%) articles included in this review. In a study published in the UK, lack of role models – mothers, other female relatives, and friends who breastfeed – was cited as one of the potential barriers for breastfeeding [36]. Some family members and friends also actively discouraged breastfeeding, while openly questioning the benefits of this practice over bottle feeding [1, 17, 40]. Breastfeeding during family gatherings or in the presence of others was also reported as a challenge for some women from ethnic minority groups in the United Kingdom and for Black women in the United States [41, 42].

The literature reported occasional instances where breastfeeding-related decisions created conflict in women’s relationships with significant others [26]. Some women noted they were pressured by their loved one to cease breastfeeding [22], especially when women continued to breastfeed 6 months postpartum [43]. Overall, the literature suggested that partners play a central role in women’s breastfeeding practices [8], although there was no consistency in the reviewed papers regarding the partners’ expressed level of support for breastfeeding.

Knowledge, especially practical knowledge about breastfeeding, was mentioned as a barrier in 17% (*n* = 10) of the papers included in this review. While health care providers were perceived as a primary source of information on breastfeeding, some studies reported that mothers felt the information provided was not useful and occasionally contained conflicting advice [1, 17]. This finding was reported across various jurisdictions, including the United States, Sweden, the United Kingdom and Netherlands, where mothers reported they had no support at all from their health care providers which made it challenging to address breastfeeding problems [26, 38, 44].

Breastfeeding in public emerged as a key barrier from the reviewed literature and was cited in 56% (*n* = 33) of the papers. Examining the experiences of breastfeeding mothers in the United States, Spencer, Wambach, & Domain [45] suggested that some participants reported feeling “erased” from conversations while breastfeeding in public, rendering their bodies symbolically invisible. Lack of designated public spaces for breastfeeding forced many women to alter their feeding in public and to retreat to a private or a more secluded space, such as one’s personal car [25]. The oversexualization of women’s breasts was repeatedly noted as a core reason for the United States women’s negative experiences and feelings of self-consciousness about breastfeeding in front of others [45]. Studies reported women’s accounts of feeling the disapproval or disgust of others when breastfeeding in public [46, 47], and some reported that women opted out of breastfeeding in public because they did not want to make those around them feel uncomfortable [25, 40, 48].

Finally, return to paid employment was noted in the literature as a significant challenge for continuation of breastfeeding [48]. Lack of supportive workplace environments [39] or inability to express milk were cited by women as barriers for continuing breastfeeding in the United States and New Zealand [39, 49].

### Supports needed to maintain breastfeeding

Due to the central role family members played in women’s experiences of breastfeeding, support from partners as well as female relatives was cited in the literature as key factors  shaping women’s breastfeeding decisions [1, 9, 48]. In the articles published in Canada, Australia, and the United Kingdom, supportive family members allowed women to share the responsibility of feeding and other childcare activities, which reduced the pressures associated with being a new mother [19, 20]. Similarly, encouragement, breastfeeding advice, and validation from healthcare professionals were identified as positively impacting women’s experiences with breastfeeding [1, 22, 28].

Community resources, such as peer support groups, helplines, and in-home breastfeeding support provided mothers with the opportunity to access help when they need it, and hence were reported to be facilitators for breastfeeding [19, 22, 33, 44]. An increase in the usage of social media platforms, such as Facebook, among breastfeeding mothers for peer support were reported in some studies [47]. Public health breastfeeding clinics, lactation specialists, antenatal and prenatal classes, as well as education groups for mothers were identified as central support structures for the initiation and maintenance of breastfeeding [23, 24, 28, 33, 39, 50]. Based on the analysis of the reviewed literature, however, access to these services varied greatly geographically and by socio-economic status [33, 51]. It is also important to note that local and cultural context played a significant role in shaping women’s perceptions of breastfeeding. For example, a study that explored women’s breastfeeding experiences in Iceland highlighted the importance of breastfeeding in Icelandic society [52]. Women are expected to breastfeed and the decision to forgo breastfeeding is met with disproval [52]. Cultural beliefs regarding breastfeeding were also deemed important in the study of  Szafrankska and Gallagher (2016), who noted that Polish women living in Ireland had a much higher rate of initiating breastfeeding compared to Irish women [53]. They attributed these differences to familial and societal expectations regarding breastfeeding in Poland [53].

Overall, the reviewed literature suggested that women faced socio-cultural pressure to breastfeed their infants [36, 40, 54]. Women reported initiating breastfeeding due to recognition of the many benefits it brings to the health of the child, even when they were reluctant to do it for personal reasons [8]. This hints at the success of public health education campaigns on the benefits of breastfeeding, which situates breastfeeding as a new cultural norm [24].

## Discussion

**Fig. 5:**
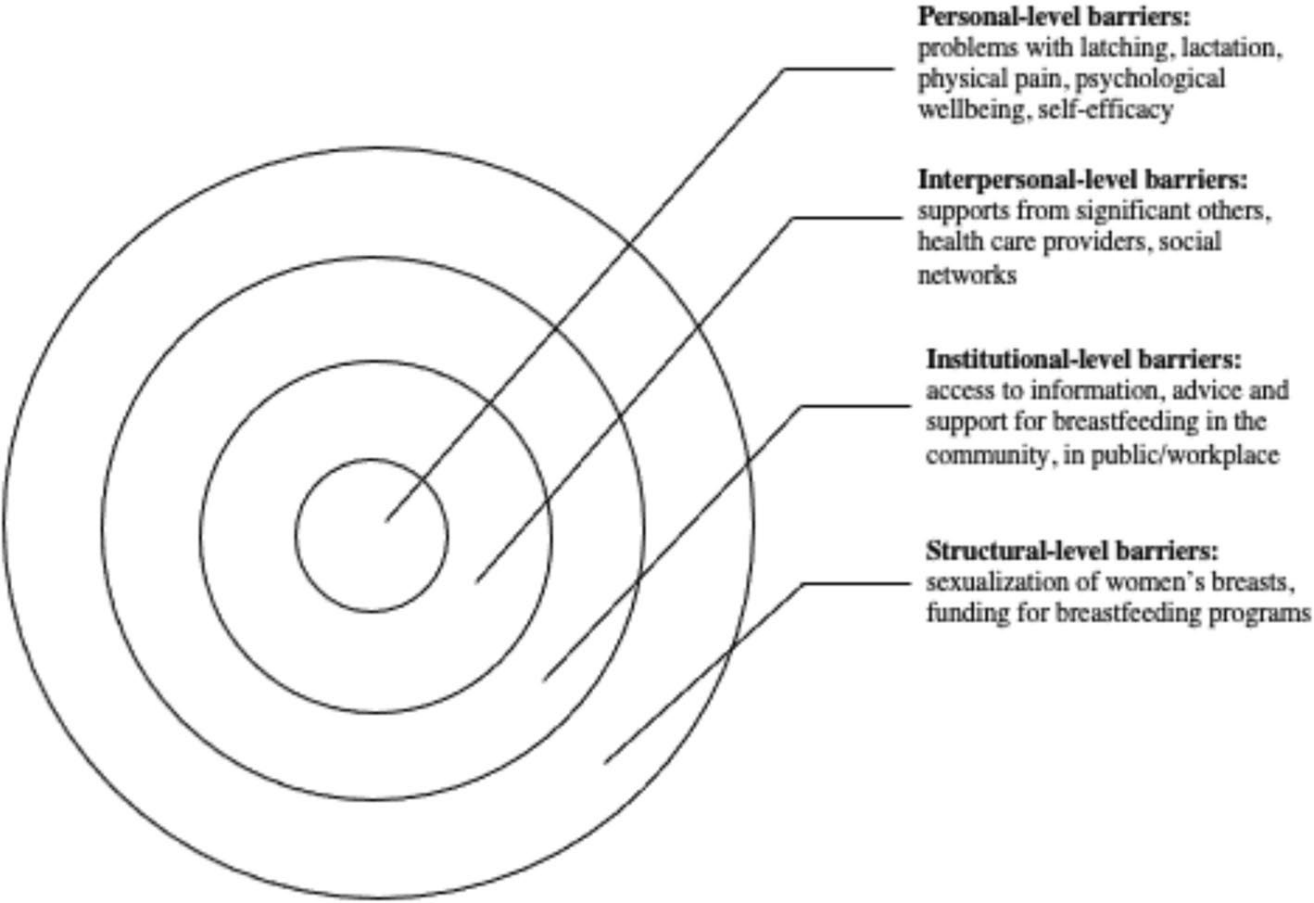
Barriers to Breastfeeding

This scoping review examined the existing empirical literature on women’s perceptions about and experiences of breastfeeding to identify how public health messaging can be tailored to improve breastfeeding rates. The literature suggests that, overall, mothers are aware of the positive impacts of breastfeeding and have strong motivation to breastfeed [37]. However, women who chose to breastfeed also experience many barriers related to their social interactions with significant others and their unique socio-cultural contexts [25]. These different factors, summarized in Fig. 5, should be considered in developing public health activities that promote breastfeeding. Breastfeeding experiences for women were very similar across the United Kingdom, United States, Canada, and Australia based on the studies included in this review. Likewise, barriers and supports to breastfeeding identified by women across the countries situated in the global north were quite similar. However, local policy context also impacted women’s experiences of breastfeeding. For example, maintaining breastfeeding while returning to paid employment has been identified as a challenge for mothers in the United States [39, 45], a country with relatively short paid parental leave. Still, challenges with balancing breastfeeding while returning to paid employment were also noticed among women in New Zealand, despite a more generous maternity leave [49]. This suggests that while local and institutional policies might shape women’s experiences of breastfeeding, interpersonal and personal factors can also play a central role in how long they breastfeed their infants. Evidently, the importance of significant others, such as family members or friends, in providing support to breastfeeding mothers was cited as a key facilitator for breastfeeding across multiple geographic locations [29, 34, 48]. In addition, cultural beliefs and practices were also cited as an important component in either promoting breastfeeding or deterring women’s desire to initiate or maintain breastfeeding [15, 29, 37]. Societal support for breastfeeding and cultural practices can therefore partly explain the variation in breastfeeding rates across different countries [15, 21]. Figure 5 summarizes the key barriers identified in the literature that inhibit women’s ability to breastfeed.

At the individual level, women might experience challenges with breastfeeding stemming from various physiological and psychological problems, such as issues with latching, perceived or actual lack of breastmilk, and physical pain associated with breastfeeding. The onset of postpartum depression or other psychological problems may also impact women’s ability to breastfeed [54]. Given that many women assume that breastfeeding will happen “naturally” [15, 40] these challenges can deter women from initiating or continuing breastfeeding. In light of these personal challenges, it is important to consider the potential challenges associated with breastfeeding that are conveyed to new mothers through the simplified message “breast is best” [21]. While breastfeeding may come easy to some women, most papers included in this review pointed to various challenges associated with initiating or maintaining breastfeeding [19, 33]. By modifying public health messaging regarding breastfeeding to acknowledge that breastfeeding may pose a challenge and offering supports to new mothers, it might be possible to alleviate some of the guilt mothers experience when they are unable to breastfeed.

Barriers that can be experienced at the interpersonal level concern women’s communication with others regarding their breastfeeding choices and practices. The reviewed literature shows a strong impact of women’s social networks on their decision to breastfeed [24, 33]. In particular, significant others – partners, mothers, siblings and close friends – seem to have a considerable influence over mothers’ decision to breastfeed [42, 53, 55]. Hence, public health messaging should target not only mothers, but also their significant others in developing breastfeeding campaigns. Social media may also be a potential medium for sharing supports and information regarding breastfeeding with new mothers and their significant others.

There is also a strong need for breastfeeding supports at the institutional and community levels. Access to lactation consultants, sound and practical advice from health care providers, and availability of physical spaces in the community and (for women who return to paid employment) in the workplace can provide more opportunities for mothers who want to breastfeed [18, 33, 44]. The findings from this review show, however, that access to these supports and resources vary greatly, and often the women who need them the most lack access to them [56].

While women make decisions about breastfeeding in light of their own personal circumstances, it is important to note that these circumstances are shaped by larger structural, social, and cultural factors. For instance, mothers may feel reluctant to breastfeed in public, which may stem from their familiarity with dominant cultural perspectives that label breasts as objects for sexualized pleasure [48]. The reviewed literature also showed that, despite the initial support, mothers who continue to breastfeed past the first year may be judged and scrutinized by others [47]. Tailoring public health care messaging to local communities with their own unique breastfeeding-related beliefs might help to create a larger social change in sociocultural norms regarding breastfeeding practices.

The literature included in this scoping review identified the importance of support from community services and health care providers in facilitating women’s breastfeeding behaviours [22, 24]. Unfortunately, some mothers felt that the support and information they received was inadequate, impractical, or infused with conflicting messaging [28, 44]. To make breastfeeding support more accessible to women across different social positions and geographic locations, it is important to acknowledge the need for the development of formal infrastructure that promotes breastfeeding. This includes training health care providers to help women struggling with breastfeeding and allocating sufficient funding for such initiatives.

Overall, this scoping review revealed the need for healthcare professionals to provide practical breastfeeding advice and realistic solutions to women encountering difficulties with breastfeeding. Public health messaging surrounding breastfeeding must re-invent breastfeeding as a “family practice” that requires collaboration between the breastfeeding mother, their partner, as well as extended family to ensure that women are supported as they breastfeed [8]. The literature also highlighted the issue of healthcare professionals easily giving up on women who encounter problems with breastfeeding and automatically recommending the initiation of formula use without further consideration towards solutions for breastfeeding difficulties [19]. While some challenges associated with breastfeeding are informed by local culture or health care policies, most of the barriers experienced by breastfeeding women are remarkably universal. Women often struggle with initiation of breastfeeding, lack of support from their significant others, and lack of appropriate places and spaces to breastfeed [25, 26, 33, 39]. A change in public health messaging to a more flexible messaging that recognizes the challenges of breastfeeding is needed to help women overcome negative feelings associated with failure to breastfeed. Offering more personalized advice and support to breastfeeding mothers can improve women’s experiences and increase the rates of breastfeeding while also boosting mothers’ sense of self-efficacy.

### Limitations

This scoping review has several limitations. First, the focus on “women’s experiences” rendered broad search criteria but may have resulted in the over or underrepresentation of specific findings in this review. Also, the exclusion of empirical work published in languages other than English rendered this review reliant on the papers published predominantly in English-speaking countries. Finally, consistent with Arksey and O’Malley’s [18] scoping review methodology, we did not appraise the quality of the reviewed literature. Notwithstanding these limitations, this review provides important insights into women’s experiences of breastfeeding and offers practical strategies for improving dominant public health messaging on the importance of breastfeeding.

## Conclusion

Women who breastfeed encounter many difficulties when they initiate breastfeeding, and most women are unsuccessful in adhering to current public health breastfeeding guidelines. This scoping review highlighted the need for reconfiguring public health messaging to acknowledge the challenges many women experience with breastfeeding and include women’s social networks as a target audience for such messaging. This review also shows that breastfeeding supports and counselling are needed by all women, but there is also a need to tailor public health messaging to local social norms and culture. The role social institutions and cultural discourses have on women’s experiences of breastfeeding must also be acknowledged and leveraged by health care professionals promoting breastfeeding.

## Data Availability

All data generated or analysed during this study are included in this published article [and its supplementary information files].
